# Supplementary material for: Probabilistic coherence, logical consistency, and Bayesian learning: Neural language models as epistemic agents
Source: PLoS One. 2023 Feb 9;18(2):e0281372. doi: 10.1371/journal.pone.0281372 (PMC9910757; doi:10.1371/journal.pone.0281372)
Supplement: S1 Appendix — Description of how degrees of belief can be assigned to non-atomic L-sequences. (PDF) [file pone.0281372.s001.pdf]

S1 Appendix. Credences of non-atomic sentences. Description of how degrees of belief can be assigned to non-atomic L-sequences.

Let us suppose that a credence function $Bel(\cdot|\cdot)$ satisfies (Reflexivity), (Commutation), (Complement), and (Multiplication) as defined in Section Popper metrics. This would allow us to introduce the degree of belief in a non-atomic L-sequence, which cannot be directly elicited, by stipulation. For if we define

$$Pr(\varphi\psi|\chi) := Pr(\varphi|\psi\chi) \times Pr(\psi|\chi),$$

then (Multiplication*) holds per definition, and (Multiplication) implies (Commutation*).

In a similar vein, we may simply stipulate that

$$Pr(\bar{s}|\chi) := 1 - Pr(s|\chi)$$

for non-atomic sequences $s \in S$. However, a function $Pr : S \times S \to [0;1]$ that satisfies (Reflexivity), (Commutation), (Multiplication) for arbitrary $\varphi, \psi, \chi \in S$, and (Complement) for atomic $a \in S$ is not necessarily extendable to a full Popper measure on the closure of S: E.g., it may violate (Reflexivity) for the complement of non-atomic sequences.
