# Supplementary material for: Probabilistic coherence, logical consistency, and Bayesian learning: Neural language models as epistemic agents
Source: PLoS One. 2023 Feb 9;18(2):e0281372. doi: 10.1371/journal.pone.0281372 (PMC9910757; doi:10.1371/journal.pone.0281372)
Supplement: S2 Appendix — Discusses Popper metrics as proxies for global probabilistic incoherence. (PDF) [file pone.0281372.s002.pdf]

S2 Appendix. Measure of probabilistic incoherence. Discusses Popper metrics as proxies for global probabilistic incoherence.

Let's consider an arbitrary credence function $Bel : \mathcal{S} \times \mathcal{S} \to [0;1]$. A theoretically elegant measure for the degree of global incoherence of $Bel(\cdot|\cdot)$ consists in the distance to the closest coherent Popper function Pr on $\mathcal{S} \times \mathcal{S}$ [1]. Formally:

$$\textsc{GlobalIncoherence}(Bel) = \min_{Pr}\big(\Delta(Bel, Pr)\big)$$

for some distance measure Δ between credence functions. Such a measure is conceptually compelling as it can be shown (for suitable choice of Δ) to be equivalent with an incoherence measure based on aggregated losses in Dutch book settings [2].

Calculating a corresponding measure amounts to an optimization problem. In the case of RANKERs' credences, however, this optimization problem is computationally challenging. Moreover, even inferring the actual complete credence function Bel through elicitation has not been feasible computationally.

Yet, the four Popper metrics can be seen as rough approximations of such a more systematic global incoherence measure:

The Popper metrics are mean squared *differences* between the equations that express the probabilistic constraints on degrees of belief. In the case of (Reflexivity), (Commutation), and (Complement), these differences, calculated for some credences Bel, represent the aggregated "credence mass" that has to be shifted so that the corresponding constraint becomes satisfied. In the case of (Multiplication), the relevant difference is one in squared credence mass. Now, these (squared) credence masses, which one has to move while revising a credence function such that it satisfies the corresponding constraints, are *proxies* for the distance to the closest coherent credence function: the less credence mass one has to shift in order to turn the original credence function into a proper probability measure, the smaller the distance between the original function and the closest coherent credence function, and hence the smaller the degree of global incoherence (GLOBALINCOHERENCE).

References

[1] Osherson D, Lane D, Hartley P, Batsell R. Coherent probability from incoherent judgment. Journal of Experimental Psychology: Applied. 2091;7(1):3.

[2] Staffel J. Measuring the overall incoherence of credence functions. Synthese. 2015;192(5):1467–1493.
