# Supplementary material for: Probabilistic coherence, logical consistency, and Bayesian learning: Neural language models as epistemic agents
Source: PLoS One. 2023 Feb 9;18(2):e0281372. doi: 10.1371/journal.pone.0281372 (PMC9910757; doi:10.1371/journal.pone.0281372)
Supplement: S3 Appendix — Motivation of the transitivity constraint for credences. (PDF) [file pone.0281372.s003.pdf]

S3 Appendix. Transitivity constraint. Motivation of the transitivity constraint for credences.

If L-sentences φ, ψ entail L-sentence χ by transitivity, i.e. $\varphi, \psi \models \chi$, we require (cf. Section Consistency metrics) that

$$Bel(\chi) \geq Bel(\varphi) \cdot Bel(\psi), \tag{Transitivity}$$

which can be derived with the probabilistic multiplication rule, and effectively expresses a fuzzy product t-norm: With $Bel(p) \geq Bel(q)$ for any sequence q that entails p, we have

$$Bel(\chi) \geq Bel(\varphi\psi) = Bel(\varphi|\psi) \cdot Bel(\psi) \tag{1}$$

and hence, **assuming independence** of φ and ψ,

$$Bel(\chi) \geq Bel(\varphi) \cdot Bel(\psi).$$

As a reviewer has pointed out, the independence assumption is of course crucial here. If, according to an agent's degrees of belief Bel, φ and ψ are negatively relevant to each other, i.e., $Bel(\varphi|\psi) < Bel(\varphi)$, then (Transitivity) might be violated although the credences are probabilistically coherent. That is why (Transitivity) is not a probabilistic, but an additional, objective constraint on RANKER's degrees of belief. The justification of the constraint hinges on the independence assumption. So why should a rational agent treat, for example, the L-sentences a_1Ra_2 and a_2Ra_3 as independent?

- The language L just consists in atomic sentences and lacks any resources to express relations between such facts. Likewise, in a model of L, the basic facts are unrelated. So there seems to be no objective justification for assuming that a_1 standing in relation R to a_2 is positively or negatively relevant for a_2 standing in relation R to a_3. Reminiscent of the principle of indifference, one might argue that, therefore, a_1Ra_2 and a_2Ra_3 should be treated as probabilistically independent.

- In the training data, L-sentences a_1Ra_2 and a_2Ra_3 are probabilistically independent. The frequency of a_1Ra_2 in the corpus has no bearing on the frequency of a_2Ra_3, which ultimately stems from the way belief systems of simulated authors are sampled. Now, one might argue, the credences of a RANKER, trained on such a corpus, should reflect the objective independence contained in data.

Alternatively, rather than justifying the independence assumption on apriori grounds, one might consider, in future research, a modification of the transitivity constraint, either by directly checking inequality (1), or by explicitly requiring, as antecedent condition when checking (Transitivity), that $Bel(\varphi|\psi) \approx Bel(\varphi)$.
