# Supplementary material for: Probabilistic coherence, logical consistency, and Bayesian learning: Neural language models as epistemic agents
Source: PLoS One. 2023 Feb 9;18(2):e0281372. doi: 10.1371/journal.pone.0281372 (PMC9910757; doi:10.1371/journal.pone.0281372)
Supplement: S1 Algorithm — (PDF) [file pone.0281372.s004.pdf]

S1 Algorithm. Text-production by simulated author.

```
Input: p_inf = .6; p_dual = .2; p_append = .95;
min_length // minimum length of text to be produced
theory // belief system of the author
reach // reach threshold of the author
Output: text
begin
    text = ← ∅ ;
    while length of text < min_length do
        if random() < p_inf then
            // Compose argument
            sequence ← sample transitivity inference with true sentences according to
              theory and within reach ;
            sequence ← randomly paraphrase sentences in sequence ;
            sequence ← randomly shuffle premises in sequence ;
            if random() < p_dual then
                sequence ← append paraphrase of conclusion to sequence
            end
        else
            // Compose fact
            sequence ← sample single true sentence according to theory and within
              reach ;
            sequence ← randomly paraphrase sequence ;
            if random() < p_dual then
                sequence ← append paraphrase of sequence to sequence
            end
        end
        if random() < p_append then
            // Append new sequence at end of text
            text ← text + sequence;
        else
            // Insert sequence earlier
            text1, text2 ← split text randomly into two lists of sentences ;
            text ← text1 + sequence + text2
        end
    end
end
```
