# Supplementary material for: Probabilistic coherence, logical consistency, and Bayesian learning: Neural language models as epistemic agents
Source: PLoS One. 2023 Feb 9;18(2):e0281372. doi: 10.1371/journal.pone.0281372 (PMC9910757; doi:10.1371/journal.pone.0281372)
Supplement: S2 Algorithm — (PDF) [file pone.0281372.s005.pdf]

S2 Algorithm. Self-training loop.

```
Input: max_steps = 600; num_texts = 200; r_pt_augm = 0.4; decoding_parameters;
for step = 1 to max_steps do
    sen_list ← ∅ ;
    // 1. generate texts and retain well-formed sentences
    for t = 1 to num_texts do
        // 1.1. prompt construction
        prompt ← construct prompt with sentences the model believes to be true ;
        // 1.2. text generation
        texts, scores ← query model with prompt (text completion task, beam sampling
          with decoding_parameters) ;
        // 1.3. split texts and retain well-formed sentences
        sentences ← split texts into sub-sequences of length 3 ;
        sentences ← remove mis-formed sentences from sentences ;
        sen_list ← append sentences and corresp. scores to sen_list
    end
    // 2. filter well-formed sentences
    sen_list ← remove sentences originating from texts with less than 6 well-formed
      sentences ;
    sen_list ← remove sentences originating from texts with score below 85th score
      percentile ;
    // 3. construct denoising training data
    train_data ← mask predicate letter in each sentence from sen_list ;
    // 4. augment self-generated data with pre-training examples
    train_data ← sample and append denoising examples from pre-training (with ratio
      r_pt_augm) ;
    // 5. train model
    model ← train model on augmented train_data for 1 epoch
end
```

decoding_parameters: To generate texts during self-training, we use beam sampling decoding as implemented by [1] with the following parameters:

number of beams	5
number of return sequences	5
do_sample	True
top_p	.7
max_length	60
no_repeat_ngram_size	3

References

[1] Wolf T, Debut L, Sanh V, Chaumond J, Delangue C, Moi A, et al. Transformers: State-of-the-Art Natural Language Processing. In: Proceedings of the 2020 Conference on Empirical Methods in Natural Language Processing: System Demonstrations. Online: Association for Computational Linguistics; 2020. p. 38–45. Available from: https://www.aclweb.org/anthology/2020.emnlp-demos.6.
