# Supplementary material for: Probabilistic coherence, logical consistency, and Bayesian learning: Neural language models as epistemic agents
Source: PLoS One. 2023 Feb 9;18(2):e0281372. doi: 10.1371/journal.pone.0281372 (PMC9910757; doi:10.1371/journal.pone.0281372)
Supplement: S3 Algorithm — (PDF) [file pone.0281372.s006.pdf]

S3 Algorithm. Prompt construction.

```
Input: len_prompt = 3 // number of sentences
prompt ← ∅ ;
a_1, a_2, a_3, a_4 ← sample (len_prompt + 1) tokens from domain D ;
for i = 1 to len_prompt do
    s ← a_i R a_{i+1} ;
    s̄ ← a_i S a_{i+1} ;
    if BEL_M(s) > BEL_M(s̄) then
        prompt ← append s to prompt ;
    else
        prompt ← append s̄ to prompt ;
    end
end
return prompt
```
