# Supplementary material for: Probabilistic coherence, logical consistency, and Bayesian learning: Neural language models as epistemic agents
Source: PLoS One. 2023 Feb 9;18(2):e0281372. doi: 10.1371/journal.pone.0281372 (PMC9910757; doi:10.1371/journal.pone.0281372)

**S1 Fig. Eval loss during pre-training.** Eval loss during pre-training, each line corresponds to one of 60 models.

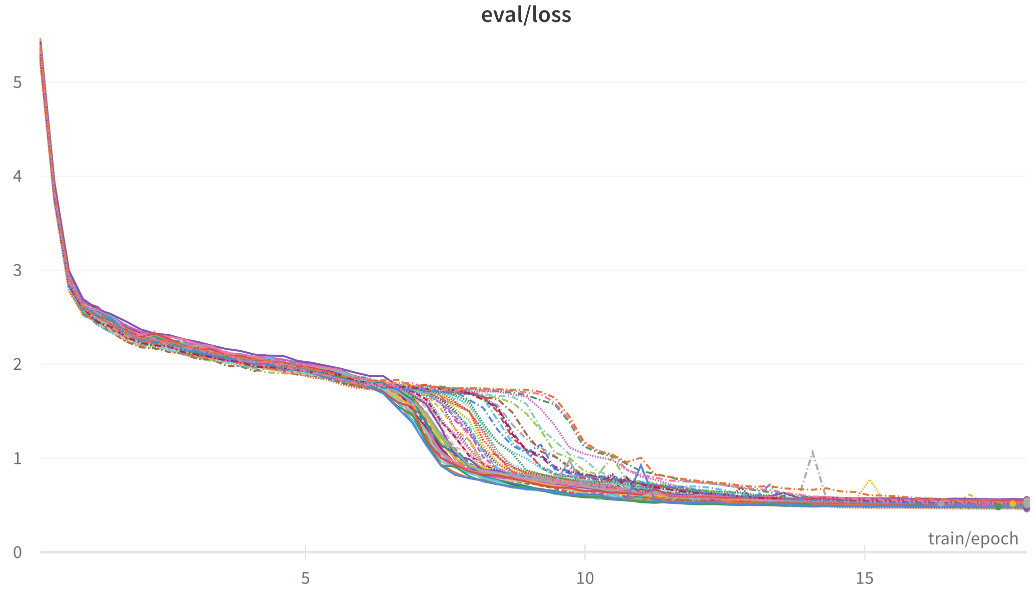

Supplement: S1 Fig — Eval loss during pre-training, each line corresponds to one of 60 models. (PDF) [file pone.0281372.s007.pdf]
