# Supplementary material for: Probabilistic coherence, logical consistency, and Bayesian learning: Neural language models as epistemic agents
Source: PLoS One. 2023 Feb 9;18(2):e0281372. doi: 10.1371/journal.pone.0281372 (PMC9910757; doi:10.1371/journal.pone.0281372)

S2 Fig. Evolution of Popper metrics *complementarity* and *reflexivity* during self-training. Left: inferentially closed pre-training corpora (reach=∞); right: inferentially incomplete pre-training corpora (reach=50). Metric evolutions are aggregated over all agents whose belief systems display a similar joint entropy (cf. bins to the very right).

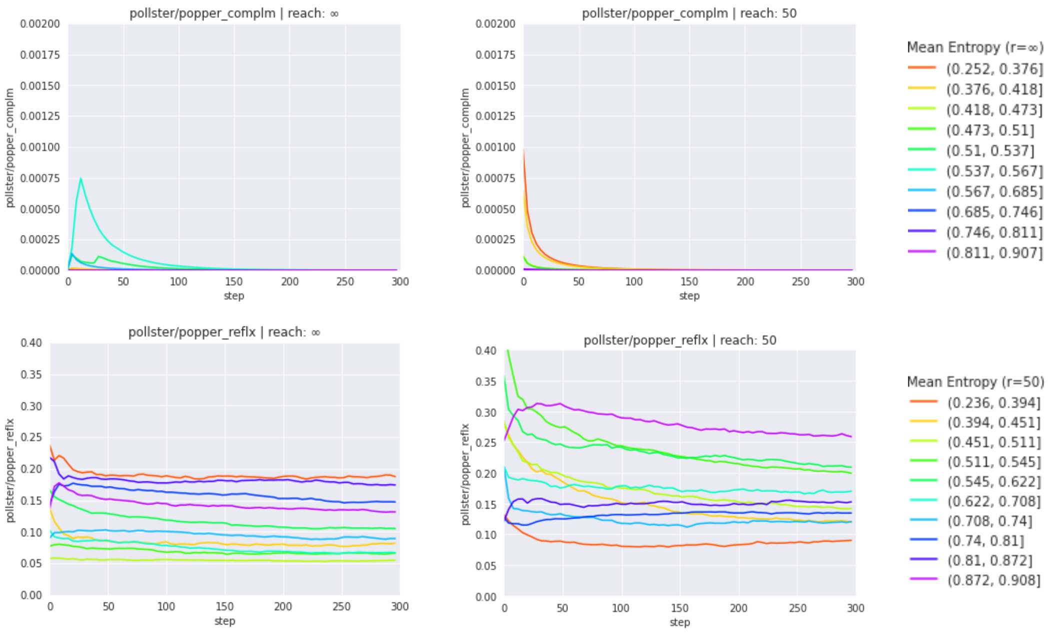

Supplement: S2 Fig — Left: inferentially closed pre-training corpora (reach=∞); right: inferentially incomplete pre-training corpora (reach = 50). Metric evolutions are aggregated over all agents whose belief systems display a similar joint entropy (cf. bins to the very right). (PDF) [file pone.0281372.s008.pdf]
