# Supplementary material for: Probabilistic coherence, logical consistency, and Bayesian learning: Neural language models as epistemic agents
Source: PLoS One. 2023 Feb 9;18(2):e0281372. doi: 10.1371/journal.pone.0281372 (PMC9910757; doi:10.1371/journal.pone.0281372)

S4 Fig. Global dynamics (evolution of joint entropy, global distance, volatility) during self-training. Left: inferentially closed pre-training corpora (reach=∞); right: inferentially incomplete pre-training corpora (reach=50). Metric evolutions are aggregated over all agents whose belief systems display a similar joint entropy (cf. legends in **S2 Fig.**).

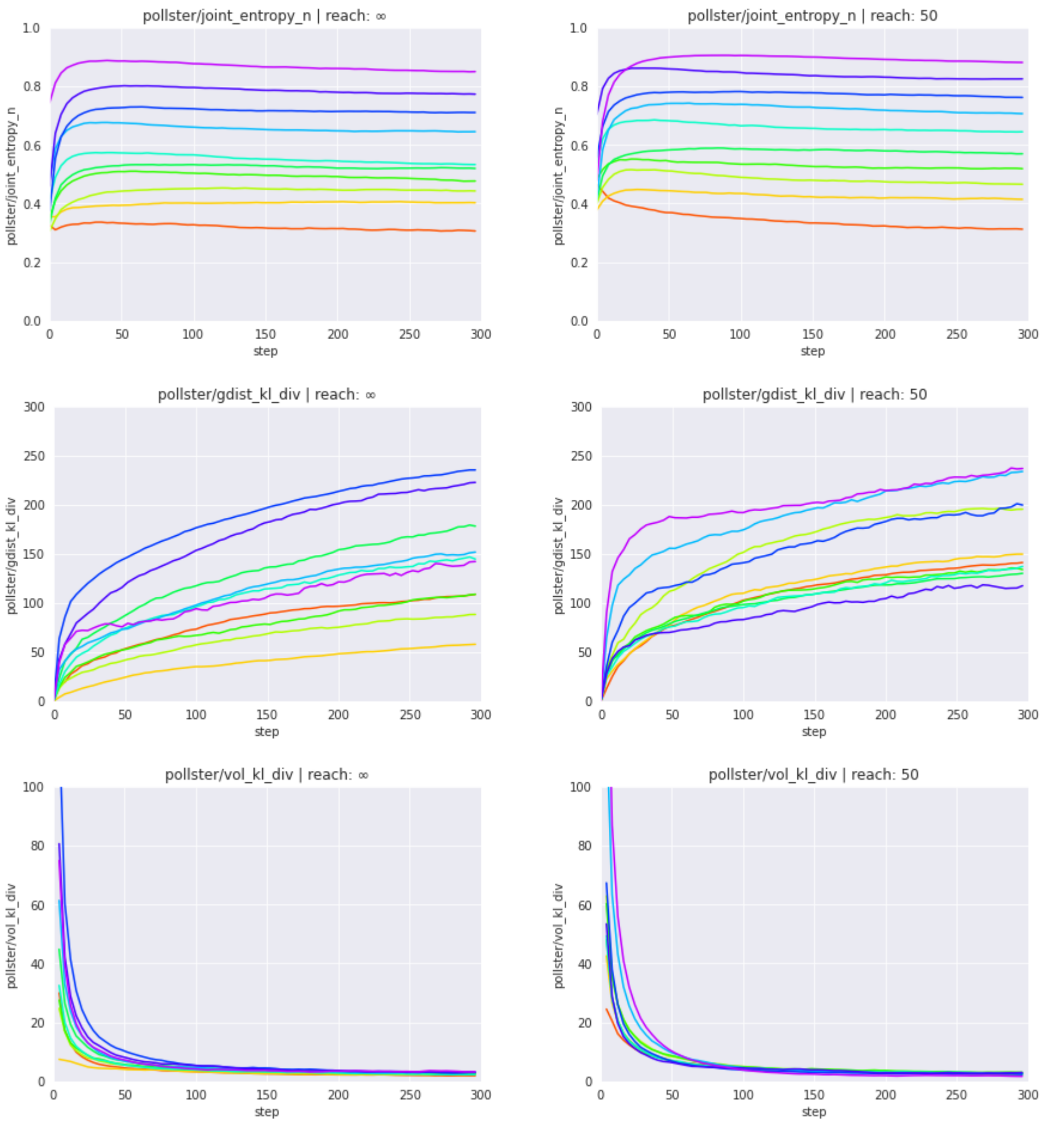

Supplement: S4 Fig — Left: inferentially closed pre-training corpora (reach=∞); right: inferentially incomplete pre-training corpora (reach = 50). Metric evolutions are aggregated over all agents whose belief systems display a similar joint entropy (cf. legends in S2 Fig). (PDF) [file pone.0281372.s010.pdf]
