# Supplementary material for: Probabilistic coherence, logical consistency, and Bayesian learning: Neural language models as epistemic agents
Source: PLoS One. 2023 Feb 9;18(2):e0281372. doi: 10.1371/journal.pone.0281372 (PMC9910757; doi:10.1371/journal.pone.0281372)

S8 Fig. Logical alignment of pretrained models. Logical alignment of pretrained models (step=0) as a function of background knowledge ratio in the training corpus (rows) and number of distractors (columns). For each model, 1000 inferences (premises, conclusion, distractors) are sampled, and for each inference, the unconditional degree of belief in the conclusion (x-axis) as well as the conditional degree of belief given premises and distractors are elicited. The y-axis show the absolute difference between conditional and unconditional beliefs.

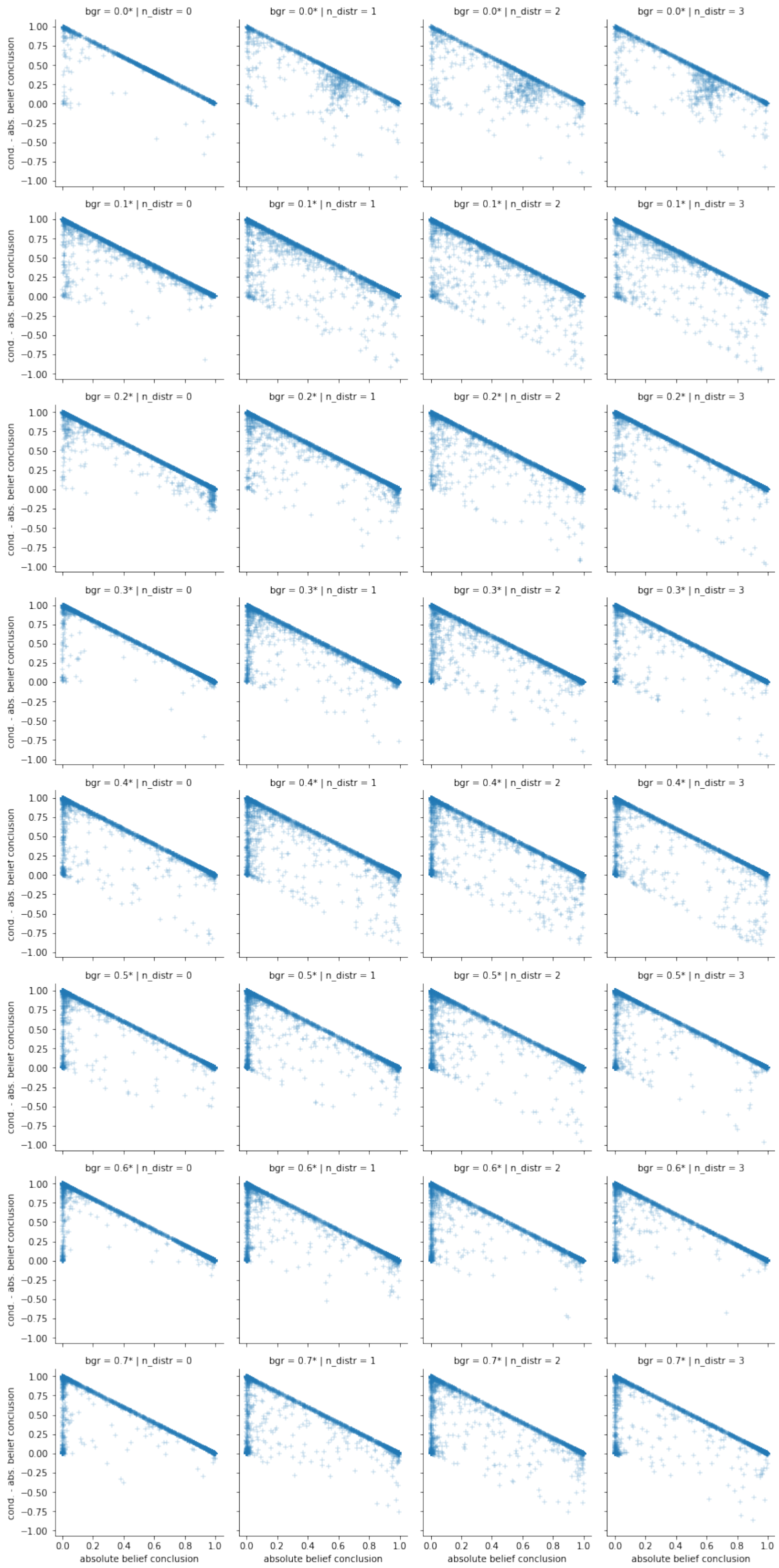

Supplement: S8 Fig — Logical alignment of pretrained models (step = 0) as a function of background knowledge ratio in the training corpus (rows) and number of distractors (columns). For each model, 1000 inferences (premises, conclusion, distractors) are sampled, and for each inference, the unconditional degree of belief in the conclusion (x-axis) as well as the conditional degree of belief given premises and distractors are elicited. The y-axis show the absolute difference between conditional and unconditional beliefs. (PDF) [file pone.0281372.s014.pdf]
