# Supplementary material for: Probabilistic coherence, logical consistency, and Bayesian learning: Neural language models as epistemic agents
Source: PLoS One. 2023 Feb 9;18(2):e0281372. doi: 10.1371/journal.pone.0281372 (PMC9910757; doi:10.1371/journal.pone.0281372)

S9 Fig. Evolution of Bayesian Metrics before and after evidence introduction. Evidence introduction regimes: "prompt" (rows 1,2); and "append_gen" (rows 3,4). Inferential closure of pre-training corpora: reach=∞ (row 1,3); reach=50 (rows 2,4). Evidential entrenchment: columns correspond to quartiles.

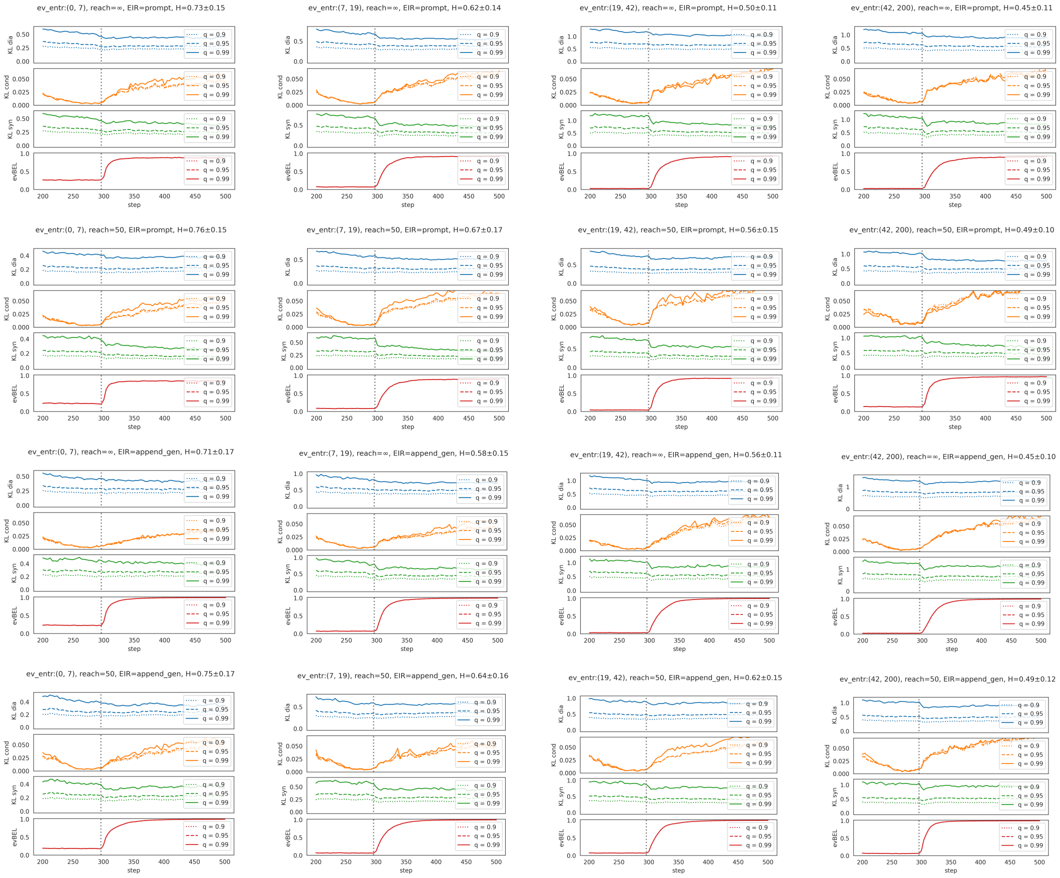

Supplement: S9 Fig — Evidence introduction regimes: “prompt” (rows 1,2); and “append_gen” (rows 3,4). Inferential closure of pre-training corpora: reach=∞ (row 1,3); reach = 50 (rows 2,4). Evidential entrenchment: columns correspond to quartiles. (PDF) [file pone.0281372.s015.pdf]
