# Supplementary material for: Probabilistic coherence, logical consistency, and Bayesian learning: Neural language models as epistemic agents
Source: PLoS One. 2023 Feb 9;18(2):e0281372. doi: 10.1371/journal.pone.0281372 (PMC9910757; doi:10.1371/journal.pone.0281372)

S10 Fig. Synchronous KL divergence before and after evidence introduction. Synchronous KL divergence (between unconditional belief and conditional belief given the evidence) before and after evidence introduction (evidence introduction regime "prompt") for 1024 individual beliefs elicited in four selected models (pre-trained on corpora with background ratio 0.5). Each row displays four different self-training runs of one and the same model.

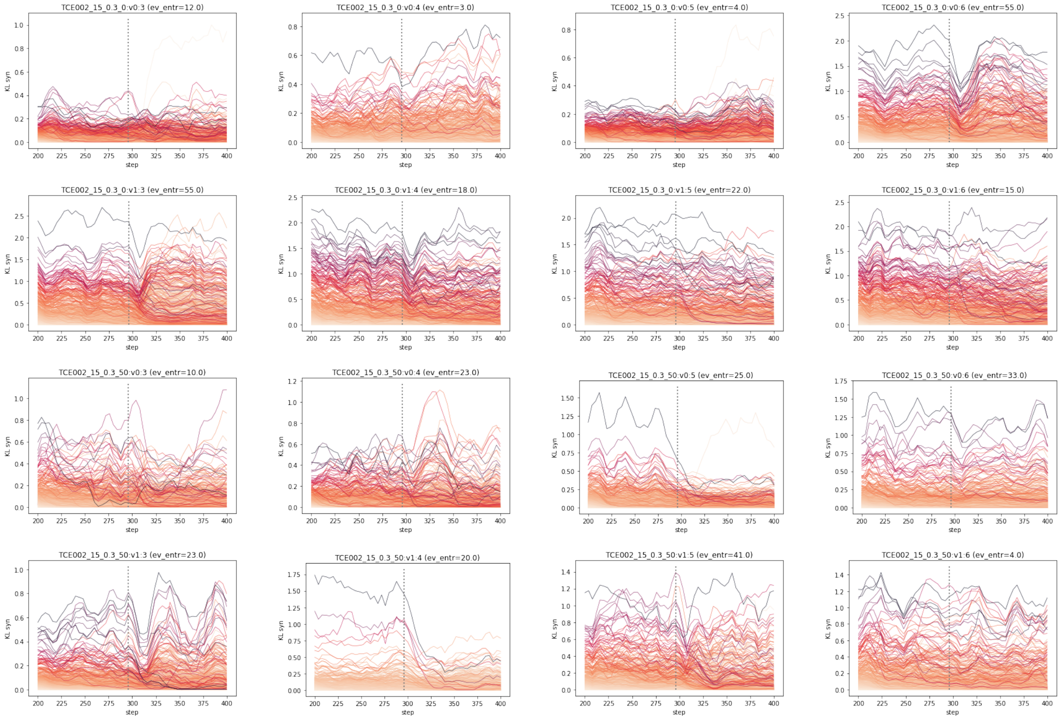

Supplement: S10 Fig — Synchronous KL divergence (between unconditional belief and conditional belief given the evidence) before and after evidence introduction (evidence introduction regime “prompt”) for 1024 individual beliefs elicited in four selected models (pre-trained on corpora with background ratio 0.5). Each row displays four different self-training runs of one and the same model. (PDF) [file pone.0281372.s016.pdf]
