# Supplementary material for: Probabilistic coherence, logical consistency, and Bayesian learning: Neural language models as epistemic agents
Source: PLoS One. 2023 Feb 9;18(2):e0281372. doi: 10.1371/journal.pone.0281372 (PMC9910757; doi:10.1371/journal.pone.0281372)

S11 Fig. Comparison of Popper Metrics before and after evidence introduction. Comparison of Popper Metrics before and after evidence introduction for different evidence introduction regimes. Left column: inferentially closed pre-training corpora (reach=∞); right column: inferentially incomplete pre-training corpora (reach=50).

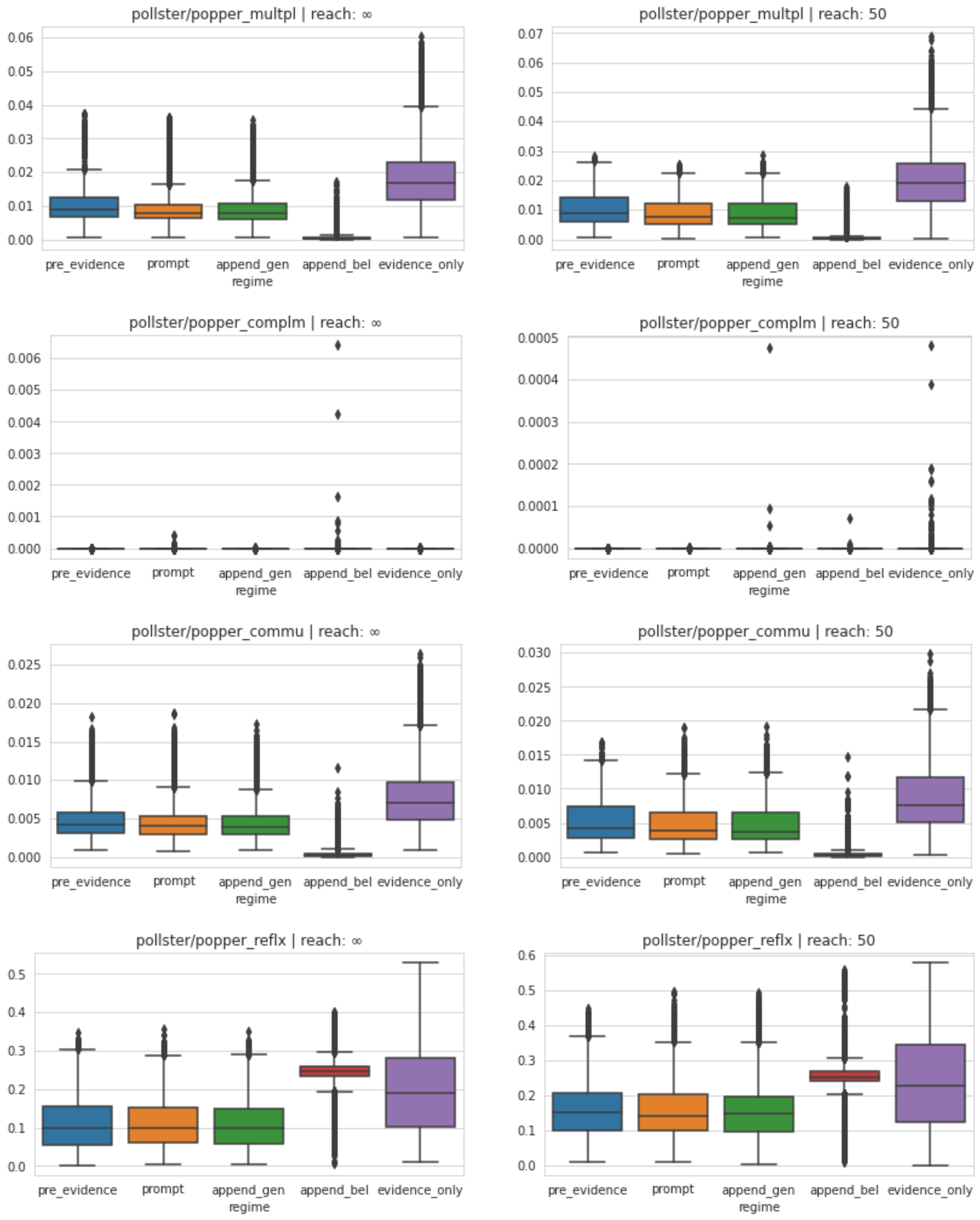

Supplement: S11 Fig — Comparison of Popper Metrics before and after evidence introduction for different evidence introduction regimes. Left column: inferentially closed pre-training corpora (reach=∞); right column: inferentially incomplete pre-training corpora (reach = 50). (PDF) [file pone.0281372.s017.pdf]
