# Supplementary material for: Probabilistic coherence, logical consistency, and Bayesian learning: Neural language models as epistemic agents
Source: PLoS One. 2023 Feb 9;18(2):e0281372. doi: 10.1371/journal.pone.0281372 (PMC9910757; doi:10.1371/journal.pone.0281372)

S13 Fig. Comparison of joint entropy and evidence belief before and after evidence introduction. Comparison of joint entropy and evidence belief before and after evidence introduction for different evidence introduction regimes. Left column: inferentially closed pre-training corpora (reach=∞); right column: inferentially incomplete pre-training corpora (reach=50).

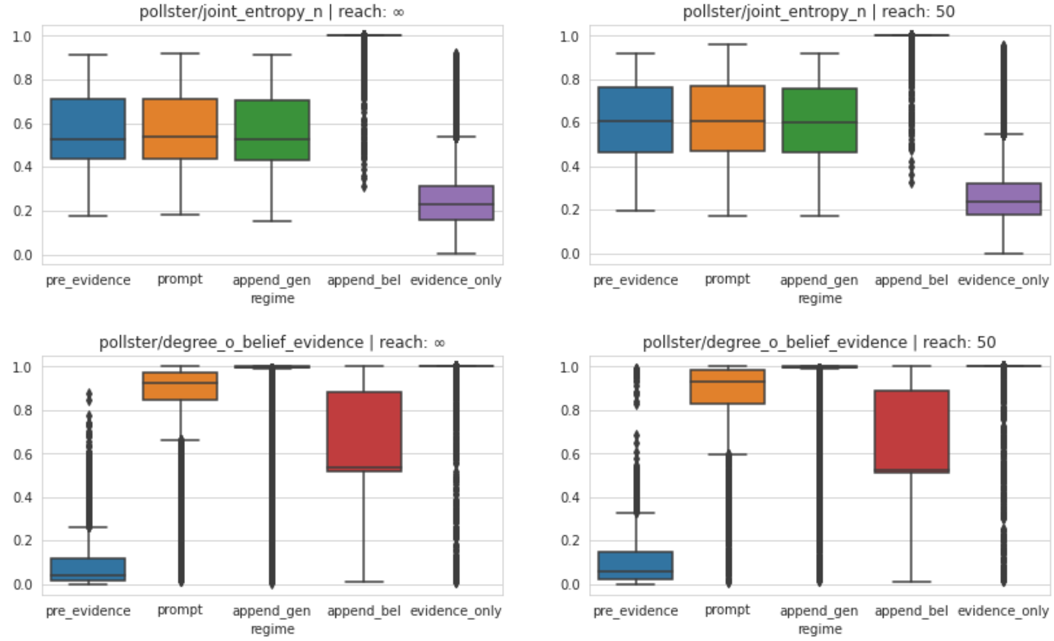

Supplement: S13 Fig — Comparison of joint entropy and evidence belief before and after evidence introduction for different evidence introduction regimes. Left column: inferentially closed pre-training corpora (reach=∞); right column: inferentially incomplete pre-training corpora (reach = 50). (PDF) [file pone.0281372.s019.pdf]
