# Supplementary material for: Probabilistic coherence, logical consistency, and Bayesian learning: Neural language models as epistemic agents
Source: PLoS One. 2023 Feb 9;18(2):e0281372. doi: 10.1371/journal.pone.0281372 (PMC9910757; doi:10.1371/journal.pone.0281372)
Supplement: S1 Table — Statistical comparison of metrics pre (steps 0–299) and post (steps 300–600) evidence introduction for evidence integration regime “prompt”: standard deviation of metric pre evidence introduction (STD PRE); difference in means post vs pre (DELTA MEAN); p-value of t-test for independence of pre and post metric samples (p); mean difference relative to standard deviation (DELTA/STD); boolean variable indicating statistically significant (p<0.05) deterioration of metric (sign). (PDF) [file pone.0281372.s020.pdf]

S1 Table. Statistical comparison of metrics pre and post evidence introduction. Statistical comparison of metrics pre (steps 0–299) and post (steps 300–600) evidence introduction for evidence integration regime ”prompt”: standard deviation of metric pre evidence introduction (STD PRE); difference in means post vs pre (DELTA MEAN); p-value of t-test for independence of pre and post metric samples (p); mean difference relative to standard deviation (DELTA/STD); boolean variable indicating statistically significant (p¡0.05) deterioration of metric (sign).

		STD PRE	DELTA MEAN	p	DELTA/STD	sign
reach	metric					
∞	popper_commu	3.09e-03	-8.54e-04	2.58e-100	-2.76e-01	F
	popper_complm	3.05e-04	-5.82e-06	6.87e-03	-1.91e-02	F
	popper_multpl	7.35e-03	-2.91e-03	8.58e-214	-3.96e-01	F
	popper_reflx	7.53e-02	-4.10e-03	1.83e-04	-5.45e-02	F
	logalgn_eqv	8.74e-03	-2.02e-04	1.35e-01	-2.32e-02	F
	logalgn_eqvcond	1.50e-02	-2.86e-03	1.11e-45	-1.90e-01	F
	logalgn_impl	7.26e-02	3.40e-03	1.21e-03	4.69e-02	T
	tr_viol_mean_mltp	1.16e-03	-1.09e-04	1.79e-27	-9.36e-02	F
	tr_viol_ratio_mltp	2.96e-02	4.34e-03	2.78e-22	1.47e-01	T
50	popper_commu	3.79e-03	-1.15e-03	4.92e-108	-3.02e-01	F
	popper_complm	7.45e-05	-2.99e-06	1.53e-08	-4.01e-02	F
	popper_multpl	7.47e-03	-3.67e-03	0.00e+00	-4.91e-01	F
	popper_reflx	1.08e-01	-1.23e-02	5.21e-17	-1.13e-01	F
	logalgn_eqv	1.32e-02	-8.23e-04	3.41e-04	-6.25e-02	F
	logalgn_eqvcond	8.91e-03	-2.28e-03	3.29e-104	-2.56e-01	F
	logalgn_impl	6.36e-02	5.52e-03	3.09e-08	8.69e-02	T
	tr_viol_mean_mltp	6.92e-03	-5.99e-04	6.11e-08	-8.66e-02	F
	tr_viol_ratio_mltp	5.61e-02	-1.51e-02	1.63e-68	-2.70e-01	F
