# Supplementary material for: Probabilistic coherence, logical consistency, and Bayesian learning: Neural language models as epistemic agents
Source: PLoS One. 2023 Feb 9;18(2):e0281372. doi: 10.1371/journal.pone.0281372 (PMC9910757; doi:10.1371/journal.pone.0281372)
Supplement: Attachment — Submitted filename: betz_richardson_coverletter_091122.pdf [file pone.0281372.s021.pdf]

PONE-D-22-15831: Submission of First Revision

Dear editors, dear reviewers,

Thank you very much for the detailed feedback on our manuscript "*Probabilistic coherence, logical consistency, and Bayesian learning: neural language models as epistemic agents*", and for the invitation to submit a revised version! We've carefully considered the issues raised, and thoroughly revised the text.

We highlight the major changes made in the point-by-point replies below. Over and above the issues raised in the reviews, we've

1. revised the formal notation so as to increase clarity of the exposition;
2. added additional references to relevant literature, e.g. regarding back-translation, or the emergence of language in deep multi-agent systems,

all of which is documented by tracking changes (blue).

Please don't hesitate to get back to us in case any issues (formal or substantial) remain unresolved!

Sincerely yours,

Gregor Betz and Kyle Richardson

Karlsruhe, 09/11/2022

Journal requirements

> 4. Thank you for stating the following in the Acknowledgments Section of your manuscript:
>
> This work is supported by the Helmholtz Association Initiative and Networking Fund on the HAICORE@KIT partition.
>
> However, funding information should not appear in the Acknowledgments section or other areas of your manuscript. We will only publish funding information present in the Funding Statement section of the online submission form. Please remove any funding-related text from the manuscript and let us know how you would like to update your Funding Statement. Currently, your Funding Statement reads as follows:
>
> This work is supported by the Helmholtz Association Initiative and Networking Fund on the HAICORE@KIT partition. The funders had no role in study design, data collection and analysis, decision to publish, or preparation of the manuscript.
>
> Please include your amended statements within your cover letter; we will change the online submission form on your behalf.

FUNDING STATEMENT: “This work is supported by the Helmholtz Association Initiative and Networking Fund on the HAICORE@KIT partition. The funders had no role in study design, data collection and analysis, decision to publish, or preparation of the manuscript.”

> 5. Thank you for stating the following in your Competing Interests section:
>
> NO
>
> Please complete your Competing Interests on the online submission form to state any Competing Interests. If you have no competing interests, please state “The authors have declared that no competing interests exist.”, as detailed online in our guide for authors at http://journals.plos.org/plosone/s/submit-now This information should be included in your cover letter; we will change the online submission form on your behalf.

COMPETING INTERESTS: “The authors have declared that no competing interests exist.”

> 6. We note that you have stated that you will provide repository information for your data at acceptance. Should your manuscript be accepted for publication, we will hold it until you provide the relevant accession numbers or DOIs necessary to access your data. If you wish to make changes to your Data Availability statement, please describe these changes in your cover letter and we will update your Data Availability statement to reflect the information you provide.

That’s fine. Technically, we have tracked the experiments and results with wandb. The data is organized in 3 wandb projects, which we’re planning to make public. So we’d have three “data repositories”. Does that work for PLOS One?

> 7. Please amend your list of authors on the manuscript to ensure that each author is linked to an affiliation. Authors’ affiliations should reflect the institution where the work was done (if authors moved subsequently, you can also list the new affiliation stating “current affiliation:....” as necessary).

We’ve done so.

> 8. Please ensure that you refer to Figure 1,2,3,4,5,6,7,8,13,14,15,16 and 17 in your text as, if accepted, production will need this reference to link the reader to the figure.

In accordance with Submission Guidelines, we’re now correctly distinguishing between main figures and SI figures.

Review 1

> This is an impressive and interesting article, making (as far as I know) novel both conceptual and technical points about the way in which the probabilistic and Bayesian coherence of NLMs may be assessed. I suspect it warrants publication, but I have a few conceptual questions that it may help for the authors to clarify before publication.

Thanks a lot for the encouragement! We reply to the six important points you raise below.

> 1) What exactly is the force of the Rationality Hypothesis? As the authors say in defense of it, it is an existential hypothesis about what a possible NLM might compute, in the right circumstances. But neural nets are Turing complete, meaning of course there will be SOME net that computes any function we like, including a function that outputs probabilistically coherent credences and adjusts them in a Bayesian fashion. So what exactly is the form of the hypothesis that is uncertain? Is the idea that such a neural net may require too different an architecture (or training regimen) from standard NLMs to count as an NLM? I think it'd help understand the point of the paper better to have a bit more discussion on this front—though obviously there are difficult issues here and I don't mean to hold the publication of the paper hostage to settling all of them perfectly clearly!
>
> Perhaps here's another way to put the conceptual worry. The authors motivate their proposal by suggesting that it bears on whether neural nets may eventually exhibit AGI, since AGI agents must be rational. But it's trivially easy to construct programs that meet all our author's metrics perfectly: simply define a (non-neural-net) program that outputs a probability function over state desriptions in a Boolean languge, and calculates probabilities of arbirary sentences by sums of their state descriptions. (Of course, this is computationally HARD, since the number of state descriptions explodes with the number of atomic sentences; but it's clearly programmable.) The fact that we can define such a Bayesian program doesn't seem to provide much if any evidence that it's possible to generate an AGI using a similar architecture. So why think the neural net case is any different?

Our paper is all about neural language models. These represent *a very specific kind* of neural net, or AI system: they are just probabilistic word prediction machines, trained on the internet as text corpus. It is much discussed in AI & NLP, what are the (potential, or actual) cognitive skills of these machines.

It is true that the questions we discuss might seem awkward/obvious when

asked for neural nets in general (like: Can some suitably trained neural net exhibit rational skills?). However, restricted to the specific class of nets we're considering here, the questions are very much unresolved and pertinent.

In the title, in the abstract, and in the first paragraph of the paper, we're referring to neural language models as objects of our investigation, so our impression was that the delineation of the research question is sufficiently clear. – But please come back to us in case you're not convinced!

> 2) Somewhat relatedly, I was surprised that the language did not contain Boolean operations. After all, that's what I'd initially expect was needed for a neural net to be "learning to be logically coherent". Why was this choice made? Is there a principled difficulty with running similar analyses with a more complex language, or is it simply an implementational difficulty? A bit more discussion would be helpful.

That's also an interesting, fairly general point. The methodological idea here is that complexity of formal or computational models is no intrinsic virtue. If you can make a point with a simple model, why use a more complex model to make the same point? Our artificial language seems just rich enough (semantically, inferentially) to answer our questions Q1-Q4 in surprising ways. This being so, having a more complex language (say, with boolean connectives, or with epistemic operators), it seems, would make sense as part of a robustness / sensitivity analysis. Maybe that's exactly what you're pointing to. Also, that's precisely what we explicitly call for in the concluding section (future research). There are nor principal difficulties in re-implementing our experiments with more complex languages. There are just much more choices to make in designing these experiments...

> 3) Why exactly does the belief elicitation protocol make sense, wherein we get the network's credences as it's probabilistic prediction for the completion of a masked string? Imagine you had a HUMAN doing this task: reading a bunch of sentences, and then predicting the next sentence. Clearly their prediction wouldn't necessarily line up with their degree of belief that the sentence is true; rather it's something closer to their degree of belief that "in this context, this corpus will present me with this string', or something like that. Is the thought that if they fully trust the corpus, that would be equivalent to their credence? I'm not sure that's right either. Difficult issues, we won't get to the bottom of them here. But more discussion for this way of measuring, and what it presupposes, would be nice.

Here, the technical details of the elicitation procedure become important. We're not eliciting credences by letting the NLM predict / generate the next sentence. We're really just masking a single token. Given the language L, there are just two permissible substitutions for the masked token (R/S), and hence it's really

a binary classification task the model engages in when we elicit its belief.

When eliciting an unconditional belief, it's as if we'd confront a human in a task where cities are compared in terms of number of inhabitants with the query: “Chicago [?] New York”, allowing the person only to choose “>” or “<”.

A related and interesting finding, documented in a similar and now published study of ours (https://www.frontiersin.org/articles/10.3389/frai.2022.900943/full), is that when generating texts, RANKERS tend to produce sentences which they actually dis-believe if these sentences follow from what they have generated before. That seems to be the main driver of rational belief revision during self-training in the very first place. It also stresses the point that we're not just eliciting beliefs by letting the NLM generate / predict the next sentence.

Does this sufficiently address your point?

> 4) The measures of deviation from probabilistic coherence are interesting, and touch on a not-huge-but-growing literature on what the proper way to define such measures is. It'd be nice to have some comparisons of the authors metrics to other ones—I suspect they will have important theoretical differences, but perhaps will not deviate too much in practice. (Though it'd be interesting if they did!) Some places to look for literature on this: - Staffel 2015, “Measuring the overall incoherence of credence functions” - Staffel and de Bona, “Graded Incoherence for Accuracy-Firsters'

Super interesting. Thanks. In designing the study, we've actually been contemplating measuring coherence in terms of distance to the closest coherent credence function. That turned out to be computationally intractable (for us, at least). There's an entirely new **Appendix S2** which relates our metrics to the literature and these conceptually more compelling measures.

> 5) I don't understand the transitivity constraint on page 18. It seems to be saying that if {A,B} entails C, then Pr(C) must be at least as great as Pr(A) * Pr(C). Right? But that is not a constraint of probabilistic coherence, and is violated whenever A and B are negatively relevant. Eg suppose we have a coin that's either biased 90% for H or biased 90% for T, and we're 50-50 on which it is. Then Pr(Heads first toss) = 0.5 and Pr(Tails second toss) = 0.5, but Pr(Heads first, Tails second) = 0.09 (if H-biased, it's 0.9 * 0.1; if T-biased, it's 0.1 * 0.9; the average of those is 0.09).
>
> The appendix mentioned something about independence, but why should that be assumed in this context?

Excellent. We're completely on the same page here. We've revised the section on Popper metrics (in particular lines 301ff.) and substantially expanded the corresponding **S3 Appendix**.

> 6) The discussion of the presentation of new evidence went by a bit

> quick for me (but perhaps I was reading quickly/less-carefully as that point, so don't put too much weight on this). Intuitively, the networks are getting new evidence at the earlier stages too—namely, when they're learning what the beliefs of the informant generating the sentences are. So conceptually I found it strange that presenting new evidence later is any different than the earlier stages. Is it just because at the earlier stage we're letting the network piggyback on the informant's logical coherence, and later we want to see if they can maintain coherence on their own? Perhaps just a word or two more on this would help tired readers understand what's happening.

Yes. At the beginning of the Section "Evidential learning" we've been describing the PHASE 3 experiments as an introduction of novel evidence statements (plural). That's misleading. One major difference to PHASE 1 (where NLMs constantly observe more or less novel evidence items) is that we confront, in PHASE 3, the NLM with a single evidence item (atomic statement) to study its reaction. We revised this accordingly.

> Anyways, thanks for the thought-provoking paper!

Thanks for the comments, really, and don't hesitate to get back in case you feel there's a point we haven't fully taken into account, yet!

Review 2

> Reviewer #2: This paper examines how well certain types of natural language models can acquire characteristics of bayesian (epistemic) rationality. The paper uses RANKERS and a simple artificial language to test properties such as general probabilistic coherence and updating by conditionalization.
>
> The results are intriguing enough for me to recommend publication, but I overall was not fond of how the paper was written. The opening few pages state broad and grand theses that are ill-defined. Indeed, the Rationality Hypothesis—nominally the main subject of the paper—is stated in vague and unclear language and not made at all clear until 4 pages later (p. 6 of the ms). Some connections the authors draw were also very underspecified. For instance, on p. 3 line 63, the authors discuss Systems 1 and 2 in psychology, and it was entirely opaque to me how this investigation related to these concepts. So, I'd like to the authors being a little less grand and a little more precise in the opening pages.

Thanks for raising this issue! On the one hand, our introductory section is clearly not what you'd want to have in a paper appearing in the proceedings of some AI or NLP conference: neither in terms of length, nor in terms of precision, nor regarding the diversity and scope of issues covered. We see that, depending on the reader's expectations, the current introduction might cause frustration.

On the other hand, we're seeing PLOS One as a place where rigorous findings can be put in broader context. The submission guidelines encourage authors to have comprehensive introductory sections. And we've also been inspired by examples like https://journals.plos.org/plosone/article?id=10.1371/journal.pone.0074516#s1, which has an introductory section with more than 3700 words.

The way out might be to make all this more transparent upfront. So, right after stating the (Rationality Hypothesis), we're saying now what the purpose of this hypothesis is, and that the more specific questions we address are detailed below. Readers might hence simply jump to Q1-Q4, skipping the more philosophical discussion. We're conceding that the Rationality Hypothesis is vague and will be made precise. Plus, we're also frank about rationality being a contested concept.

With these revisions, we hope to better manage reader expectations and to allow readers to skip parts of the text they are not interested in.

> I have some reservations as well about the conclusions. For example, the authors use an extremely limited artificial language and then present the Ranker with fully coherent text and probe its partial beliefs. It isn't especially surprising if a system with such an artificial language that is presented a variety of opposing, but fully consistent passages ends up in the convex hull of the views it's presented with. While the results are interesting, I think the significance is a bit limited.

Right. This observation concerns the results of PHASE 1 of the experiments. And we agree that those are probably the least relevant findings. However, we also learn, from PHASE 1, that training on consistent texts doesn't guarantee to arrive at a consistent belief state (whether in the convex hull or not). What's really interesting, in our view, are the results from PHASE 2 and 3, which address questions Q2–Q3. That's why the paper focuses on those findings.
